# Cardiac Impairment Evaluated by Transesophageal Echocardiography and Invasive Measurements in Rats Undergoing Sinoaortic Denervation

**DOI:** 10.1371/journal.pone.0087935

**Published:** 2014-05-14

**Authors:** Raquel A. Sirvente, Maria C. Irigoyen, Leandro E. Souza, Cristiano Mostarda, Raquel N. La Fuente, Georgia O. Candido, Pamella R. M. Souza, Alessandra Medeiros, Charles Mady, Vera M. C. Salemi

**Affiliations:** 1 Cardiomyopathy Unit of the Heart Institute (InCor) do Hospital das Clínicas da Faculdade de Medicina da Universidade de São Paulo, São Paulo, Brazil; 2 Hypertension Unit of the Heart Institute (InCor) do Hospital das Clínicas da Faculdade de Medicina da Universidade de São Paulo, São Paulo, Brazil; 3 Federal University of São Paulo, Biosciences Department, Santos, São Paulo, Brazil; Universidade Federal do Rio de Janeiro, Brazil

## Abstract

**Background:**

Sympathetic hyperactivity may be related to left ventricular (LV) dysfunction and baro- and chemoreflex impairment in hypertension. However, cardiac function, regarding the association of hypertension and baroreflex dysfunction, has not been previously evaluated by transesophageal echocardiography (TEE) using intracardiac echocardiographic catheter.

**Methods and Results:**

We evaluated exercise tests, baroreflex sensitivity and cardiovascular autonomic control, cardiac function, and biventricular invasive pressures in rats 10 weeks after sinoaortic denervation (SAD). The rats (n = 32) were divided into 4 groups: 16 Wistar (W) with (n = 8) or without SAD (n = 8) and 16 spontaneously hypertensive rats (SHR) with (n = 8) or without SAD (SHRSAD) (n = 8). Blood pressure (BP) and heart rate (HR) did not change between the groups with or without SAD; however, compared to W, SHR groups had higher BP levels and BP variability was increased. Exercise testing showed that SHR had better functional capacity compared to SAD and SHRSAD. Echocardiography showed left ventricular (LV) concentric hypertrophy; segmental systolic and diastolic biventricular dysfunction; indirect signals of pulmonary arterial hypertension, mostly evident in SHRSAD. The end-diastolic right ventricular (RV) pressure increased in all groups compared to W, and the end-diastolic LV pressure increased in SHR and SHRSAD groups compared to W, and in SHRSAD compared to SAD.

**Conclusions:**

Our results suggest that baroreflex dysfunction impairs cardiac function, and increases pulmonary artery pressure, supporting a role for baroreflex dysfunction in the pathogenesis of hypertensive cardiac disease. Moreover, TEE is a useful and feasible noninvasive technique that allows the assessment of cardiac function, particularly RV indices in this model of cardiac disease.

## Introduction

The arterial baroreflex (ABR) system is one of the most powerful and rapidly acting mechanisms for controlling blood pressure (BP) and heart rate (HR). Also, it has a prognostic value in many cardiovascular diseases. In arterial hypertension, sympathetic hyperactivity seems to be related to baro- and chemoreflex impairment and left ventricular (LV) dysfunction. One method of studying ABR dysfunction is the interruption of ABR by sinoaortic denervation (SAD) [1]. Previous studies have shown that traditional SAD may induce persistent high blood pressure variability (BPV) and mild LV hypertrophy, without an increase in mean BP [2]–[3]. Also, in this model, right ventricular (RV) hypertrophy secondary to pulmonary hypertension has been shown [4]. This could be caused by hypoxic pulmonary vasoconstriction, in response to hypoventilation-induced alveolar hypoxia, which possibly resulted from carotid body chemoreceptor denervation.

In the case of spontaneously hypertensive rats (SHR), which is a genetic model of hypertension, this disease has been accompanied by moderate BPV and severe LV hypertrophy. However, a relevant issue not previously shown in SHR that have undergone SAD is the noninvasive determination of detailed cardiac function, particularly RV function, the use of tissue Doppler indices, and the indirect estimation of pulmonary artery pressure.

Transesophageal echocardiography (TEE) can be performed in animals by using an intracardiac echocardiographic catheter (ICE) and may be useful for a comprehensive cardiac evaluation in small animal echocardiography [5]–[7]. Thus, the objective of our study was to evaluate the influence of denervation in SHR by noninvasive and invasive methods of cardiac function. In our study we found that baroreflex dysfunction could be related to the pathogenesis of hypertensive cardiac disease and TEE is feasable to demonstrate the cardiac compromising in small animals with hypertensive disease.

## Methods

Experiments were performed in 2 month of age adult male Wistar (W) and spontaneously hypertensive rats (SHR) obtained from the Animal House of the University of São Paulo, São Paulo, Brazil. The rats (n = 32) were divided into 4 groups: 16 Wistar (W) with (n = 8) or without SAD (n = 8) and 16 SHR with (n = 8) or without SAD (SHRSAD) (n = 8). Ten weeks after SAD, cardiac function was evaluated by exercise testing (ET) at day 1, TEE and transthoracic (TTE, day 2), femoral artery and vein catheters were inserted for direct measurements of BP, HR, and drug administration at day 3, hemodynamic and autonomic measurements were performed at day 4 and 5, invasive evaluation of biventricular pressures were obtained at day 6, animal sacrifice and the heart was removed at day 7. Rats were housed in standard cages and fed with standard laboratory chow and water ad libitum. The animals were housed in collective polycarbonate cages in a temperature-controlled room (22°C) with a 12-hour dark-light cycle (light 07∶00–19∶00 h). The experimental protocol was approved by the institutional Animal Care and Ethics Committee of the University of São Paulo Medical School, and this investigation was conducted in accordance with the previously described ethical principles in animal research adopted by the Brazilian College of Animal Experimentation (COBEA) and the principles of the Declaration of Helsinki.

### 1. Sinoaortic Denervation

SAD was performed with the rats anesthetized with Ketamine (Parke-Davis, Brazil; 50 mg/kg, ip) and Xylazine (Bayer, Brazil; 12 mg/kg, ip). A 3-cm midline incision was made, and the sternocleidomastoid muscles were reflected laterally, exposing the neurovascular sheath [1]. The common carotid arteries and the vagal trunk were isolated, and the aortic depressor fibers either traveling with the sympathetic nerve or as an isolated aortic nerve were cut. The communicating branch of the aortic fibers was also resected. The third contingent of aortic baroreceptor fibers traveling with the inferior laryngeal nerve was interrupted by resection of the superior laryngeal nerve after the carotid bifurcation had been exposed extensively for carotid stripping. To complete the procedure, the sinus nerve, all carotid branches, and the carotid body were resected [1]. After surgery, all rats were given a single dose of penicillin (10 000 U/kg, im). The W and SHR were not submitted to fictitious surgery.

### 2. Maximal Exercise Test

Maximal exercise test was performed on a treadmill (Imbrasport, Porto Alegre, Brazil) using a protocol as previously described [11]–[12]. All animals were adapted to the exercise test procedure with light exercise (e.g. 0.3 km/h; 10 min/day) for 1 week. Forty-eight hours later the animals were submitted to a maximal progressive exercise test on a motor treadmill. All maximal protocol tests started with 0.3 km/h and increased 0.3 km/h each 3 min until the animals were unable to run further, as described in previous studies [13].

### 3. Echocardiography

#### 3.1. Transesophageal echocardiography

**Figure 1:**
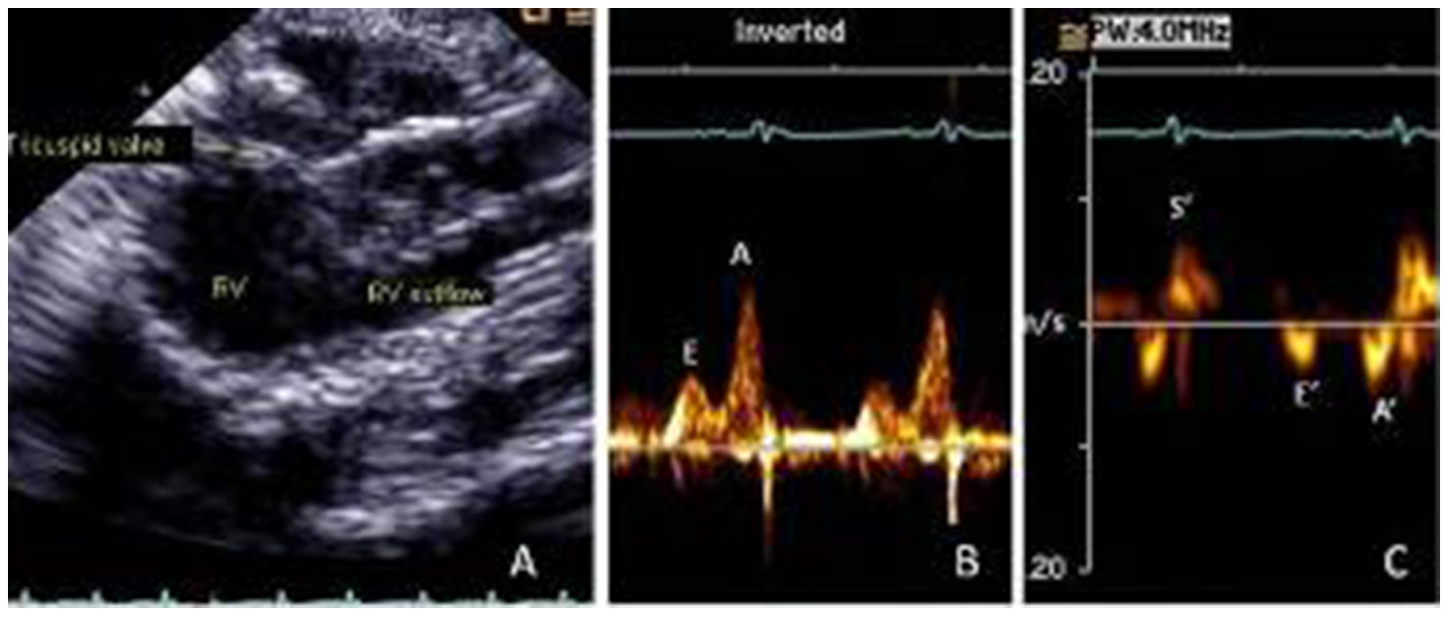
Transesophageal echocardiography of spontaneously hypertensive rats showing right ventricle (RV) (A), tricuspid inflow (B) and tissue Doppler imaging of the tricuspid annulus (C).

The animals were anesthetized with Ketamine (Parke-Davis, Brazil; 50 mg/kg, ip) and Xylazine (Bayer, Brazil; 12 mg/kg, ip), had their chest shaved, were intubated and placed on small-animal ventilator model 683 (Harvard Apparatus, Holliston, MA, USA). Heart rate was evaluated by simultaneous electrocardiographic monitoring. After lubrication, a monoplane ICE (AcuNav, Siemens, Mountain View, CA, USA) with 10 F and multiple frequency of 5.5–10 MHz was inserted into the esophagus, posterior to the left atrium. This catheter has a transducer at its tips and generates M-mode, 2-dimensional, spectral Doppler and color Doppler images [5]–[7]. It was then connected to a Sequoia 512 echocardiographic machine (Siemens, Mountain View, CA, USA). Apical 4-chamber, 2-chamber, and 3-chamber views, and RV chamber and outflow images were acquired [14]. The sequence of echocardiographic examination was 2-dimensional apical views, color Doppler and pulsed-wave Doppler of the following variables: mitral inflow [peak velocity of early (E) and late (A) waves, E/A ratio, deceleration time of E wave, atrial filling fraction (AFF) obtained by velocity time integral of A wave/velocity time integral of mitral inflow], isovolumic relaxation time (IVRT) measured from aortic valve closure to the onset of mitral inflow, and LV outflow velocity measured just below the aortic valve, from an apical 5-chamber view. Tissue Doppler imaging (TDI) of the LV septum and lateral mitral annulus: systolic velocity (S’), early velocity (E’), late velocity (A’), and E’/A’ ratio [15]. Also, RV maximum and minimum volume obtained by 2-dimensional echocardiography and RV ejection fraction (EF) was then calculated. Tricuspid inflow and TDI of the lateral tricuspid annulus were obtained, as described above for LV indices. Figure 1 shows RV diastolic dysfunction in SHR with tricuspid inflow E<A (B) and TDI tricuspid annulus E’< A’ (C). Acceleration time (AcT) and duration of the RV outflow Doppler velocity profile (TT) and its ratio were obtained. Global cardiac function was evaluated by using the myocardial performance index (MPI), which is the ratio of total time spent in isovolumic activity (isovolumic contraction time and isovolumic relaxation time) to the ejection time (ET) [16]. These Doppler time intervals were measured from the mitral inflow and LV outflow time intervals. Interval “a”, from the cessation to the onset of mitral inflow, is equal to the sum of the isovolumic contraction time, the ET and the isovolumic relaxation time. Ejection time “b” is derived from the duration of the LV outflow Doppler velocity profile. The MPI was calculated with the formula (a − b)/b. Also, MPI was obtained by TDI at the septum and lateral mitral annulus, and lateral tricuspid annulus. The advantage of these measurements over the conventional MPI is that TDI permits the evaluation of time intervals at the same cycle. All measurements were based on the average of 3 consecutive cardiac cycles. All study images were stored digitally and on VHS tapes.

#### 3.2. Transthoracic echocardiography

Transthoracic echocardiography was performed in all the animals after TEE, with a Sequoia 512 machine (Siemens, Mountain View, CA) equipped with a 10–13 MHz multifrequency linear transducer, as previously described [17]–[18]. Images were obtained with the transducer placed on each animal’s shaved chest. To obtain a more distinctive image display, a transmission gel was used between the transducer and the animal’s chest (General Imaging Gel, ATL, Reedsville, PA). Wall thickness and LV dimensions were obtained from a short-axis view at the level of the papillary muscles by M-mode echocardiography [19]. LV mass was calculated by using the following formula, previously validated in rats [20]:
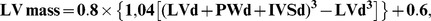
where LVd is LV end-diastolic diameter, PWd is end-diastolic posterior wall thickness, and IVSd is end-diastolic interventricular septum thickness. In addition, the relative wall thickness (RWT), which is expressed by:

**RWT = **2×PWd/LVd, was calculated.

LV fractional shortening (FS), was calculated as:
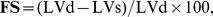
where LVs is LV end-systolic diameter.

Acceleration time (AcT) and TT of the RV outflow and its ratio were obtained at parasternal short-axis view at the level of aorta/left atrium.

### 4. Hemodynamic and Autonomic Measurements

Ten weeks after SAD, animals were anesthetized with Ketamine (Parke-Davis, Brazil; 50 mg/kg, ip) and Xylazine (Bayer, Brazil; 12 mg/kg, ip); femoral artery and vein catheters (PE-lO) filled with 0.06 mL saline solution were inserted for direct measurements of BP, HR, and drug administration. The rats were conscious and allowed to move freely in the cage during the experiments. Twenty-four hours after catheterization, the arterial catheter was connected to a transducer (Blood Pressure XDCR, Kent© Scientific, Litchfield, CT), and BP signals were recorded in conscious, freely moving rats, over a 30-minute period by a microcomputer equipped with an analog-to-digital converter board (Windaq, 2000 Hz sampling frequency, Dataq Instruments, Inc, Akron, OH). The recorded data were analyzed on a beat-to-beat basis to quantify changes in mean BP and HR. Both HR variability (HRV) and BPV were analyzed in time (SD PI = standard deviation of pulse interval, SD SBP = systolic blood pressure, PIV = pulse interval variance, SBPV = SBP variability) and frequency domains (spectral analysis) [8].

For baroreflex assessment, increasing doses of phenylephrine (0.25 to 32 ug/kg) and sodium nitroprusside (0.05 to 1.6 ug/kg) were given as sequential bolus injections (0.1 mL) to induce either an increase or decrease in BP, ranging from 5 to 40 mm Hg, and subsequent bradycardic or tachycardic responses to these BP changes [9]. The corresponding peak reflex change in HR was recorded for each dose of drug, and baroreflex sensitivity was calculated by a mean index relating changes in HR to changes in mean BP [8]–[10]. Both HR variability (HRV) and BPV were analyzed in time and frequency domains [8]. All 30-minute time series of pulse intervals and systolic BP were cubic spline interpolated (250 Hz) and decimated to be equally spaced in time. Following linear trend removal, power spectral density was obtained by fast Fourier transformation using Welch’s method^8^ over 16 384 points with a Hanning window (512) and 50% overlapping. Spectral power for very low frequency (VLF; 0–0.20 Hz), low frequency (LF; 0.20–0.75 Hz), and high frequency (HF; 0.75–3.0 Hz) bands were calculated by power spectrum density integration within each frequency bandwidth using a customized routine (MATLAB 6.0; Mathworks, Natick, MA, USA) [8].

### 5. Invasive Evaluation of Biventricular Pressures

Left ventricular and RV functions were measured invasively in anesthetized rats (pentobarbital sodium, 40 mg/kg). A polyethylene P50 catheter was inserted in the jugular vein to the RV, and signs of end-systolic (ESP) and end-diastolic pressures (EDP) of the RV were recorded for 5 minutes on a microcomputer equipped with a data acquisition system, at a sampling frequency of 2000 Hz per channel (Codas, DATAQ Instruments, Akron, OH, USA) [21]–[22]. Also, one catheter of PE-50 was inserted into the right carotid artery and advanced into the LV, and EDP of the LV, +dP/dt, -dP/dt and time constant of relaxation (Tau) were obtained.

### 6. Statistical Analysis

All data are expressed as mean ± SD. Comparisons of normally distributed continuous variables were performed with 2-way analysis of variance (ANOVA) then followed by Student-Newman-Keuls and Kruskal-Wallis tests for multiple comparisons. The Pearson or Spearman correlation coefficient was used to analyze the relation between noninvasive and invasive indices. Normality was determined by the Shapiro-Wilks test. P<0.05 was considered statistically significant. Statistical analysis was performed with SPSS v 17 (SPSS Inc. Chicago, IL, USA).

## Results

### 1. Baroreflex Sensitivity and Cardiovascular Autonomic Control

Blood pressure and HR did not change between the groups with and without SAD, although SHR had higher BP levels compared with W. BP variability was increased in SHR groups compared to W. After SAD, BP variability increased in all groups compared to W (W: 15 mmHg^2^; *SAD: 49 mmHg^2^; *SHR: 60 mmHg^2^; *SHRSAD: 137 mmHg^2^, *p<0.05 vs. W, Table 1. As expected, hypertension induces impairment of bradycardic and tachycardic responses elicited by the increased and decreased BP by using vasoactive drugs. On the other hand, SAD reduced HR baroreflex-mediated responses in both normotensive and hypertensive animals, indicating the efficacy of denervation procedure. In addition, there was no baroreflex sensitivity difference between denervated animals (SAD and SHRSAD).

**Table 1:** Blood pressure, heart rate, baroreflex sensitivity and cardiovascular autonomic modulation of normotensive and hypertensive groups.

Variables	Group			
	W	SAD	SHR	SHRSAD
HR (bpm)	334±10	327±8	347±6	358±9
PI (ms)	180±5	183±4	173±3	167±4
SD PI (ms)	9±0.6	7±0.4^a^	8±0.8	7±0.9^a^
SBP (mmHg)	126±4	119±3	190±4^a^ ^,b^	193±9^a^ ^,b^
SD SBP(mmHg)	4±3	7±0.8^a^	7±0.6^a^	11±1^a^
PIV (ms^2^)	91±11	52±7	70±13	60±12
LF (ms^2^)	2.0±0.9	3±0.9	12±3^a^ ^,b^	9±0.8^a^ ^,b^
HF (ms^2^)	10±1	9±0.7	30±6^a^ ^,b^	25±6^a^ ^,b^
LF band (%)	17±1	23±6	27±4^a^	28±2^a^
HF band (%)	82±1	76±6	72±4^a^	72±2^a^
LF/HF	0.22±0.02	0.35±0.1	0.41±0.08	0.34±0.05
SBPV (mmHg^2^)	16±2	50±11	60±10	137±28^a^ ^,b,c^
LF SBP(mmHg^2^)	3±0.6	6±2	14±2^a^ ^,b^	20±5^a^ ^,b^
BRI (bpm/mmHg)	2±0.09	0.7±0.04^a^ ^,c^	1±0.06^a^	0.6±0.03^a^ ^,c^
TRI (bpm/mmHg)	3.8±0.2	0.49±0.08^a^ ^,c^	1.29±0.15^a^	0.54±0.06^a^ ^,c^

Va1ues are mean ± SD.

W = Wistar; SAD = sinoaortic denervation; SHR = spontaneously hypertensive rats; SHRSAD = spontaneously hypertensive rats with sinoaortic denervation.

HR = heart rate; PI = pulse interval; SD PI = standard deviation of pulse interval; SBP = systolic blood pressure; SD SBP = standard deviation of systolic blood pressure; PIV = pulse interval variance; LF (ms^2^) = low-frequency band of heart rate variability; HF (ms^2^) = high-frequency band of heart rate variability; LF band (%) = low-frequency band of heart rate variability; HF band (%) = high-frequency band of heart rate variability; SBPV = SBP variability; LF SBP (mmHg^2^) = low frequency band of SBPV; BRI = bradycardic response index; TRI = tachycardic response index;

a p<0.05 vs. W, ^b^p<0.05 vs. SAD, and ^c^p<0.05 vs. SHR.

### 2. Maximal Exercise Test

Exercise test showed that the maximal velocity in the SHR group (1.4±0.3 Km/h) was increased compared to that in SAD (0.9±0.2 km/h). Similarly, SHRSAD (1.0±0.3 km/h) compared to SHR had decreased maximal velocity (1.4±0.3 km/h, p<0.05).

The W group had values intermediate between the other groups, did not have low values as the SAD and SHRSAD, nor high values as SHR, but did not differ between groups (1.16±0.11 km/h).

### 3. Echocardiography

#### 3.1 Transesophageal data

RV maximal volume increased in SHR compared to W and SAD, and RV minimal volume increased in SHR and SHRSAD compared to W (Table 2). Right ventricular EF decreased in SHRSAD compared to W (Table 2).

**Table 2:** Volumes and ejection fraction of the right ventricle, and biventricular diastolic function data obtained by transesophageal echocardiography.

Variables	W	SAD	SHR	SHRSAD
RV maximal volume (mL/kg)	1.50±0.14	1.51±0.18	1.76±0.22^a^ ^,b^	1.62±0.15
RV minimal volume (mL/kg)	0.75±0.32	0.85±0.16	1.04±0.15^a^	1.07±0.12^a^
**LV diastolic**	**function**			
E/A	1.99±0.50	1.38±0.44	2.29±0.88^b^	1.81±0.31
DT (ms)	40±4	47±8	43±7	47±8
IVRT (ms)	25±1	27°±2	37±5^a^ ^,b^	31±6^a^ ^,c^
AFF	0.25±0.04	0.37±0.10^a^	0.25±0.05^b^	0.31±0.04
**RV diastolic**	**function**			
E/A	1.67±0.35	1.00±0.42	1.06±0.68	1.44±0.76
DT (ms)	47±11	57±17	59±12	63±21
AFF	0.27±0.05	0.48±0.10^a^	0.44±0.11^a^	0.39±0.14

Va1ues are mean ± SD.

W = Wistar; SAD = sinoaortic denervation; SHR = spontaneously hypertensive rats; SHRSAD = spontaneously hypertensive rats with sinoaortic denervation.

FE VD = right ventricular ejection fraction; E/A = ratio of peak velocity of E and A waves of mitral or tricuspid inflow; DT = deceleration time of E wave; IVRT = isovolumic relaxation time of LV; AFF = atrial filling fraction.

a p<0.05 vs. W, ^b^p<0.05 vs. SAD and ^c^p<0.05 vs. SHR.

LV diastolic function showed that SHR and SHRSAD increased IVRT compared to W. Also, SAD presented increased AFF compared to W (Table 2). RV diastolic function increased AFF in SAD and SHR compared to W (Table 2).

Tissue Doppler imaging showed that S’ of the septum was reduced in SHRSAD compared to SAD and SHR. At the lateral mitral annulus, SHRSAD presented decreased S’ compared to W. At the tricuspid annulus, SHRSAD showed diminished S’ compared to W and SAD (Table 3). Also, the E’septum was reduced in SHRSAD compared to SHR. SAD augmented A’ compared to W, whereas in SHR and SHRSAD it was reduced compared to SAD (Table 3).

**Table 3:** Tissue Doppler imaging data of mitral and tricuspid annulus data obtained by transesophageal echocardiography.

Variables	W	SAD	SHR	SHRSAD
**Mitral annulus - septum**				
S’ (m/s)	0.048±0.015	0.064±0.019	0.054±0.013	0.038±0.008^b,c^
E’ (m/s)	0.039±0.010	0.045±0.013	0.049±0.008	0.038±0.007^c^
A’ (m/s)	0.026±0.005	0.043±0.015^a^	0.029±0.007^b^	0.025±0.008^b^
E’/A’	1.50±0.44	1.129±0.34	1.726±0.37	1.679±0.77
**Mitral annulus – lateral region**				
S’ (m/s)	0.055±0.015	0.046±0.011	0.041±0.011	0.036±0.014^a^
E’ (m/s)	0.035±0.01	0.039±0.015	0.034±0.011	0.035±0.009
A’ (m/s)	0.028±0.008	0.036±0.021	0.028±0.008	0.026±0.011
E’/A’	1.23±0.20	1.24±0.52	1.35±0.68	1.49±0.58
**Tricuspid annulus – lateral region**				
S’ (m/s)	0.060±0.018	0.058±0.014	0.047±0.011	0.035±0.009^a^ ^,b^
E’ (m/s)	0.042±0.010	0.044±0.012	0.038±0.006	0.036±0.008
A’ (m/s)	0.033±0.008	0.044±0.012	0.037±0.013	0.026±0.007^b^
E’/A’	1.28±0.26	1.08±0.45	1.15±0.48	1.48±0.56

Va1ues are mean ± SD.

W = Wistar; SAD = sinoaortic denervation; SHR = spontaneously hypertensive rats; SHRSAD = spontaneously hypertensive rats with sinoaortic denervation.

S’ = peak velocity of systolic velocity obtained by tissue Doppler imaging; E’ = peak velocity of early diastolic velocity obtained by tissue Doppler imaging; A’ = peak velocity of late diastolic velocity obtained by tissue Doppler imaging.

a p<0.05 vs. W, ^b^p<0.05 vs. SAD, and ^c^p<0.05 vs. SHR.

Myocardial performance index increased in SHR compared to SAD. Also, MPI TDI of the lateral region of the mitral annulus increased in SHRSAD compared to SHR (Table 4).

**Table 4:** Myocardial performance index, right ventricular outflow by transesophageal, transthoracic echocardiography and invasive data.

Variables	W	SAD	SHR	SHRSAD
**Pulsed-wave Doppler**				
LV MPI	0.47±0.18	0.39±0.14	0.60±0.15^b^	0.54±0.16
RV MPI	0.35±0.08	0.34±0.15	0.35±0.17	0.47±0.12
**Pulsed-wave tissue Doppler**				
Septum MPI	1.08±0.48	0.98±0.38	0.78±0.19	0.96±0.51
Lateral LV MPI	1.39±0.23	1.02±0.36	0.85±0.32	1.30±0.56^c^
Lateral RV MPI	1,17±0.33	0.94±0.25	0.85±0.24	1.05±0.28
**Right Ventricular outflow data TEE**				
AcT (ms)	40±2.16	33±1.35^a^	32±1.03^a^	28±1.48^a^ ^,b^
AcT/TT	0.47±0.04	0.40±0.02	0.35±0.03^a^	0.32±0.02^a^ ^,b^
**Right Ventricular outflow data TTE**				
AcT (ms)	41±3	32±3^a^	32±3^a^	26±4^a^ ^,b,c^
AcT/TT	0.37±0.03	0.30±0.04^a^	0.29±0.05^a^	0.25±0.04^a^ ^,b,c^
**Invasive data**				
ESP RV (mmHg)	31.9±3.4	42.7±7.8	38.9±16.7	45.4±10.7^a^
EDP RV (mmHg)	3.03±0.39	4.7±0.52^a^	6.61±1.16^a^ ^,b^	7.8±0.87^a^ ^,b^
EDP LV (mmHg)	5.83±0.19	8.98±1.2^a^	12.51±4.73^a^	14.57±2.52^a^ ^,b^
−dp/dt RV (mmHg/s)	−1120.7±37.8	−783.5±4.9^a^	−780.7±32.3^a^	−691.8±24.5^a^
+dp/dt RV (mmHg/s)	1553.9±71.8	100.4±30.7^a^	1259.8±18.3^a^ ^,b^	1228.1±29^a^ ^,b^
Tau RV	16.28±0.42	23.14±0.79^a^	19.37±0.49^a^ ^,b^	25.37±0.62^a^ ^,c^
−dp/dt LV (mmHg/s)	−7424.2±206.2	−4824.1±206.2^a^	−7037.5±181.8^b^	−5530.7±192^a^ ^,c^
+dp/dt LV (mmHg/s)	9169.3±138.6	6021.2±138.6^a^	9273.7±122.2^b^	8510.8±129.6^a^ ^,b,c^
Tau LV	15.28±0.94	20±0.94^a^	22±0.83^a^	24.25±0.88^a^

Va1ues are mean ± SD.

W = Wistar; SAD = sinoaortic denervation; SHR = spontaneously hypertensive rats; SHRSAD = spontaneously hypertensive rats with sinoaortic denervation.

MPI = myocardial performance index; LV = left ventricle; RV = right ventricle; AcT = aceleration time of right ventricular outflow; TT = time from the beginning to the end of right ventricular outflow; EDPRV = end-diastolic pressure of right ventricle; EDPLV = end-diastolic pressure of left ventricle; ESPRV = end-systolic pressure of the right ventricle.

a p<0.05 vs. W, ^b^p<0.05 vs. SAD, and ^c^p<0.05 vs. SHR.

All groups had a reduction of AcT compared to W and SHR, reflecting a possible increase in pulmonary artery pressure in all groups (Table 4).

#### 3.2. Transthoracic data

The amount of data gathered from M-mode echocardiography suggests concentric hypertrophy in SHR and SHRSAD compared to the other groups (Table 5). Left atrium/weight increased in SHRSAD compared to SAD (Table 5).

**Table 5:** Cardiac morphology obtained by M-mode transthoracic echocardiography.

Variables	W	SAD	SHR	SHRSAD
**Body weight (g)** **Left ventricle**	351±13	401±44	304±21^a^ ^,b^	283±13^a^ ^,b^
LVd/weight (mm/kg)	1.84±0.30	1.71±0.17	1.71±0.19	1.79±0.44
LVs/weight (mm/kg)	0.80±0.39	0.85±0.14	0.73±0.22	0.74±0.42
IVSd/weight (mm/kg)	0.41±0.03	0.39±0.03	0.65±0.10^a^ ^,b^	0.61±0.08^a^ ^,b^
PWd/weight (mm/kg)	0.43±0.03	0.42±0.04	0.65±0.10	0.60±0.22
FS (%)	58±14	50±6.7	58±11	59±15
CI (mL/min/kg)	1.43±0.55	0.81±0.48	1.63±1.15	1.01±0.40
LVMass/weight (g/kg)	3.06±0.29	2.95±0.29	3.71±0.48^a^ ^,b^	3.54±0.43^a^ ^,b^
RWT	0.48±0.09	0.49±0.03	0.77±0.14^a^ ^,b^	0.76±0.27^a^ ^,b^
**Left atrium**				
Ao (mm)	0.35±0.05	0.37±0.03	0.34±0.04	0.33±0.04
LA (mm)	0.37±0.07	0.40±0.03	0.35±0.04	0.35±0.02
LA/weight (mm/kg)	1.06±0.20	1.03±0.15	1.16±0.15	1.23±0.10^b^
Ao/LA	0.96±0.13	0.93±0.06	0.96±0.09	0.94±0.11

Va1ues are mean ± SD.

W = Wistar; SAD = sinoaortic denervation; GSHR = spontaneously hypertensive rats; GSHRDSA = spontaneously hypertensive rats with denervation;

LVd = left ventricular end-diastolic dimension; LVd = left ventricular end-systolic dimension; IVSd = interventricular septum thickness in diastole; PWd = posterior wall thickness in diastole; FS = fractional shortening; CI = cardiac index; LVMass = left ventricular mass; RWT = relative wall thickness; Ao = aortic dimension; LA = left atrium thickness in diastole.

a p<0.05 vs. W and ^b^p<0.05 vs. SAD.

Different from TEE, TTE showed that all groups had both reduced TAc and AcT/TT compared to W (Table 4).

### 4. Invasive Measurements and Relation of Noninvasive and Invasive Data

Biventricular EDP was increased in all groups compared to W (Table 4). Also, end-systolic pressure was increased in SHRSAD compared to the W group. -dP/dt RV and +dP/dt RV was decreased in all groups compared to W. Tau RV was increased in SHRSAD compared to all groups, except in SAD. –dP/dt LV and +dP/dt LV were decreased in SHRSAD and SAD compared to all groups. Tau LV was increased in SHRSAD compared to all groups, except SHR (Table 4).

There was a negative correlation between AcT of RV outflow velocity and RV EDP (r = 0.75; p = 0.005), a positive correlation between RV maximal volume and LV mass/weight (r = 0.5; p = 0.0016), and a positive correlation between AcT of RV outflow by TTE and TEE (r = 0.53; p = 0.001).

### 5. Relation between Invasive and Echocardiographic Indices

We performed the relation between invasive and echocardiographic indices (Table 6).

**Table 6:** Correlations between invasive and noninvasive data in all animals.

Right Ventricular Indices		
Comparison	Correlation coefficient (r)	P value
−dP/dt vs AFF	0.5985	P = 0.0016
−dP/dt vs E/E’	−0.5594	P = 0.0016
−dP/dt vs AcT/TT TTE	−0.6953	P<0.0001
−dP/dt vs AcT TTE	−0.8462	P<0.0001
−dP/dtvs AcT TEE	−0.6449	P = 0.0002
−dP/dt vs TT	−0.6724	P<0.0001
+dP/dt vs AFF	−0.5010	P = 0.0107
+dP/dt vs AcT TTE	0.5421	P = 0.0035
EDP vs E/E’	−0.5703	P = 0.0012
EDP vs TT	−0.5275	P = 0.0033
EDP vs AcT/TT	−0.7341	P<0.0001
EDP vs AcT TEE	−0.6080	P = 0.0005
EDP vs AFF	0.4258	P = 0.0338
EDP vs AcT TTE	−0.8302	P<0.0001
Tau vs AcT/TT	−0.7263	P<0.0001
Tau vs E/E’	−0.5732	P = 0.0014
Tau vs TT	−0.4164	P = 0.0275
Tau vs AcT TTE	−0.8117	P<0.0001
Tau vs AcT TEE	−0.4768	P = 0.0103
**Left Ventricular Indices**		
**Comparison**	**Correlation coefficient (r)**	**P value**
−dP/dt vs MPI	−0.3627	P = 0.0489
+dP/dt vs MPI	0.4892	P = 0.0061
+dP/dt vs LV weight	0.4318	P = 0.0396
+dP/dt vs LVMass/weight	0.4913	P = 0.0050
EDP vs CI	−0.4716	P = 0.0150
EDPvs EF	−0.6141	P = 0.0007
EDP vs LVMass	0.5459	P = 0.0018
EDP vs LV	0.5160	P = 0.0117
Tau vs EF	−0.6148	P = 0.0006

Values are mean ± SD.

AFF = atrial filling fraction; LV = left ventricle; AcT TTE = aceleration time of right ventricular outflow (transthoracic echocardiography); AcT TEE = aceleration time of right ventricular outflow (transesophageal echocardiography); TT = time from the beginning to the end of right ventricular outflow; CI = cardiac index; LVMass = left ventricular mass; MPI = myocardial performance index; EF = ejection fraction; E/E’ =  ratio of peak velocity of E wave of mitral inflow and peak velocity of early diastolic velocity obtained by tissue Doppler imaging; EDP = end-diastolic pressure.

## Discussion

In the present study, we found that 10 weeks after SAD, baroreflex sensitivity impairment significantly changed the morphological, functional, and hemodynamic cardiac variables in W and SHR. Also, denervated animals did not have altered BP and HR, but did have increased BP variability and decreased functional capacity. Also, baroreflex dysfunction participated in RV impairment, first demonstrated noninvasively here, by transesophageal echocardiography.

Krieger first described SAD in rats in 1964, and this technique has been useful to assess the baroreflex role in both physiological and pathological situations [1]–[23]; [24]. In the first description, this model was considered to be neurogenic hypertension, but it is currently regarded as a BP variability model, because as shows hypertension in the acute phase (24 h), followed by tachycardia, but with a return to normal hemodynamic values in the chronic phase [25]. Likewise, the renal sympathetic hyperactivity detected in the acute phase returns to normal in the chronic phase. Nevertheless, increased BP variability occurs in chronic SAD [6]. These findings confirm the usefulness of SAD as a model of BP variability and baroreflex dysfunction. In our study, the denervated groups did not have altered basal BP and HR, and maintained the augmented BP variability. Additionally, W groups did not have a difference in sympathetic modulation, assessed by LF of HR in SAD and controls. On the other hand, SHR had an increase in this component and kept it augmented after SAD compared to the control group. Both groups had decreased PI variability and increased SBP variability. The latter was increased in SAD groups, and was additionally augmented in SHRSAD. Baroreflex sensitivity was decreased in SHR groups, and additionally reduced in SAD, with/without hypertension. Our findings highlight the level of autonomic dysfunction seen in these animals that probably contributed to the low performance during maximal exercise testing of SHRSAD compared to SHR. In addition to the limitation of the autonomic system to promote the necessary regulations during exercise test, possibly contributed to reducing the efficacy of the cardiovascular system [26]. It is important to emphasize that the peripheral control of sympathetic activity could be influenced not only by baroreceptor impairment, but also by concomitant aortic and carotid chemoreceptor impairment, characteristic of conventional SAD [24]. The evidence of autonomic dysfunction (increased sympathetic tonus, decreased baroreflex sensitivity, and augmented BP variability) may also explain the structural alterations in SHR and SAD groups. The increase in BP variability by interruption of aortic and carotid afferents, relieving the sympathetic from tonic inhibition, has been associated with target organ lesions, more than the constantly elevated BP itself has [24].

In our study, SHR with/without SAD had increased LV mass, relative wall thickness, indicating concentric LV hypertrophy [27]. In addition, there was a decrease in S’ of the septum and lateral wall mitral annulus in SHRSAD compared to SHR and SAD groups, reflecting LV systolic segmental dysfunction, even in animals with LV preserved global systolic function. This is the first study to evaluate regional systolic and diastolic biventricular function by TEE in small denervated animals.

LV diastolic function showed that only IVRT was different between groups, which was greater in SHR with/without denervation compared to W, reflecting LV diastolic dysfunction in hypertension. This parameter was reduced in SHRSAD compared to SHR. This finding could indicate advanced diastolic dysfunction, as LA size was increased in SHRSAD, but not in SAD. A previous study from our group showed that diastolic function was worse in denervated normotensive animals, independent of BP variability [28].

Left ventricular global myocardial function by pulsed-wave MPI was increased in SHR compared to W, as expected. However, the same index analyzed by LV TDI was increased in SHRSAD compared to SHR, suggesting that baroreflex dysfunction could induce an additional impairment in this index. The importance of our findings is that the MPI has prognostic value in many cardiopathies, including pulmonary artery hypertension [29], which could allow the future use of this index for baroreflex dysfunction and its functional repercussion of the heart. Another advantage of this method is the high reproducibility and the fact that it is not based on geometric assumptions of ventricular morphology [30]. In our study, TDI MPI may have produced a more consistent finding, because, with this method, both systolic and diastolic functions are evaluated in the same cardiac cycle.

Different from our previous study, we did not find LV morphological alterations in SAD in the present study. This finding could be related to the denervation process, which is not always homogeneous in all animals, as some afferent fibers have a different path to the central nervous system, via the vagal nerve, for instance.

Considering the high BPV variability in hypertension, in SAD and the association of both, RV hypertrophy has been described [16], although not ever found. Van Vliet [4] reported RV hypertrophy, but this was associated with a reduction in body weight, which could falsely increase RV mass. In addition, in normotensive rats after SAD, Miao *et al* found an increase in RV mass of 22% and a decrease in 6% in body weight (unpublished data).

RV assessed by TEE showed increased maximum and minimum volumes in hypertensive animals, denervated or not, suggesting that hypertension associated or not with LV dysfunction may be related to RV dysfunction. Although previous guidelines have suggested that the RV area, and not volume, be measured, here we studied the volumes that could also reflect RV alterations, as they were obtained by 2-dimensional echocardiography [12]. In addition, there was a positive correlation between LV mass and RV volume, supporting the previous hypothesis. Moreover, RV function was reduced in SHRSAD, indicating greater compromise in this group.

End-diastolic RV pressure was greater in experimental groups compared to W, indicated that SAD with/without hypertension was related to right ventricular diastolic dysfunction.

Possibly, the decrease in acceleration time of RV outflow in SAD, SHR, and SHRSAD indicates increased pulmonary artery pressure and its consequent RV hypertrophy, especially because the denervation included the resection of the afferent chemoreceptors. Moreover, we agree with Van Vliet [4] hypotheses, indicating that in SAD the RV hypertrophy develops secondary to afferent chemoreceptor denervation, leading to consequences in pulmonary circulation and RV hypertrophy. In accordance with this, AcT obtained by TTE and TEE, associated with a decrease in RV ejection fraction and S’ of tricuspid annulus, reinforces the indication of a possible increased pulmonary pressure in SHRSAD. Also, we found a good correlation of AcT between TEE and TTE (r = 0.53, p = 0.001). Our results showed that invasive parameters of systolic and diastolic RV and LV function are more compromised in SHRSAD, thus, validating the noninvasive data (Table 4).

## Conclusions

We found that baroreflex dysfunction impairs biventricular and LA functions, and increases pulmonary artery pressure, particularly in hypertensive rats, supporting a role for baroreflex dysfunction in the pathogenesis of hypertensive cardiac disease. Also, TEE is a useful and feasible noninvasive technique allowing the assessment of cardiac function, particularly RV indices in this model of cardiac disease. Our data was supported by concomitant invasive measurements.

### Limitations of the Study

It was necessary to perform orotracheal intubation to perform TEE, because respiratory depression occurred by the manipulation of the TEE transducer in the esophagus. In humans, the assessment of RV volumetric quantification and the EF measurements have many limitations, because the complex RV shape limits performance of the required assessments [30]. This is even more challenging in small animals. In our study, the use of TEE allowed the acquisition of some RV conventional echocardiographic views, but we could not obtain all views frequently obtained in comprehensive evaluation of the right heart. Using TEE, we could detect change in global RV volumes in hypertensive groups (with/without SAD), but the position of the septum did not change. In addition, using TTE to analyze tricuspid inflow and TDI of the lateral tricuspid annulus in small animals is also challenging. But in our study, the use of TEE allowed this assessment.
