# Correction: Cardiac Impairment Evaluated by Transesophageal Echocardiography and Invasive Measurements in Rats Undergoing Sinoaortic Denervation

**DOI:** 10.1371/journal.pone.0105744

**Published:** 2014-08-07

**Authors:** The PLOS ONE Staff

The following information is missing from the Funding section: This study was supported by CNPq grant 485757/2007-9.
